# Matrix Metalloproteinases and Their Multiple Roles in Alzheimer's Disease

**DOI:** 10.1155/2014/908636

**Published:** 2014-06-24

**Authors:** Xiang-Xiang Wang, Meng-Shan Tan, Jin-Tai Yu, Lan Tan

**Affiliations:** ^1^Department of Neurology, Qingdao Municipal Hospital, School of Medicine, Qingdao University, No. 5 Donghai Middle Road, Qingdao 266071, China; ^2^Department of Neurology, Qingdao Huangdao District People's Hospital, Qingdao 266400, China; ^3^Department of Neurology, Qingdao Municipal Hospital, College of Medicine and Pharmaceutics, Ocean University of China, Qingdao 266003, China

## Abstract

Alzheimer's disease (AD) is the most prevalent type of dementia. Pathological changes in the AD brain include amyloid-*β* (A*β*) plaques and neurofibrillary tangles (NFTs), as well as neuronal death and synaptic loss. Matrix metalloproteinases (MMPs) play an important role as inflammatory components in the pathogenesis of AD. MMP-2 might be assumed to have a protective role in AD and is the major MMP which is directly linked to A*β* in the brain. Synthesis of MMP-9 can be induced by A*β*, and the enzymes appear to exert multiple effects in AD in senile plaque homoeostasis. The proaggregatory influence on tau oligomer formation in strategic brain regions may be a potential neurotoxic side effect of MMP-9. MMP-3 levels are correlated to the duration of AD and correlate with the CSF T-tau and P-tau levels in the elderly controls. Elevated brain levels of MMP-3 might result in increased MMP-9 activity and indirectly facilitate tau aggregation. At present, the clinical utility of these proteins, particularly in plasma or serum, as potential early diagnostic biomarkers for AD remains to be established. More research is needed to understand the diverse roles of these proteases to design specific drugs and devise therapeutic strategies for AD.

## 1. Introduction

Alzheimer's disease (AD) is the most prevalent type of dementia. Deposition of improperly processed A*β* peptides has been suggested to be the main causative factor for AD. Several lines of evidence indicate that there may be an inflammatory component to the pathology of AD. Matrix metalloproteinases (MMPs) remodel the pericellular environment by regulating the cleavage of extracellular matrix proteins, cell surface components, neurotransmitter receptors, and growth factors. The confused abilities of several MMPs to degrade amyloid precursor protein (APP) leading to aggregation of A*β*, as well as the increased expression of MMPs in postmortem brain tissue of AD patients, indicating that MMPs play an important role in the pathogenesis of AD. Their activities are determined through the induction of transcription by inflammatory mediators, through posttranslational modification by free radicals or cytokines and through inhibitory proteins such as tissue inhibitors of metalloproteinases (TIMPs) [1].

As a part of a larger super family of metzincins, MMPs are Zn^2+^ and Ca^2+^ dependent endopeptidases. Subdivided on the basis of protein-domain structure and substrate preference, metalloproteinases constitute a family of at least 28 MMPs, which can all degrade various components of the extracellular matrix (ECM). MMPs can be further divided into six main subgroups [2]. Gelatinases (such as MMP-2, 9) degrade molecules in the basal lamina around capillaries, enable angiogenesis and neurogenesis, participate in inducing cell death, and play a prominent role in injury and repair. Stromelysins (such as MMP-3, 10, 11) metabolise components of the extracellular matrix, but not the triple helical fibrillar collagens. Collagenases (such as MMP-1, 8, 13, 18) degrade triple helical fibrillar collagens of bones and cartilage. Membrane-type MMPs (MT-MMPs, MT1, 2, 3, 4, 5, 6-MMP) act at the cell surface and have several functions, including activation of other proteases and growth factors. Matrilysines (such as MMP-7) act on collagen type IV, glycoproteins, and gelatin. Other uncategorized MMPs (such as MMP-12, 19, 20, 23, 27, 28) act on amelogenin, aggrecan, and elastin [2]. Several MMPs, including all of the membrane-anchored versions, contain a furin motif (MMP-11 and MT1, 2, 3-MMP, among others) that mediates intracellular activation of the proteinase prior to its appearance outside of the cell. The activation of the other MMPs requires extracellular proteolytic processing of the secreted zymogens into the active enzymes, a process performed by MMPs or specific proteinases [1]. Most MMPs are secreted as pro-MMP, an inactive zymogen that has an autoinhibitory propeptide in its N terminus and is proteolytically activated by serine proteases or some activated MMPs. The tasks and effects of MMPs are complex; their beneficial effects include neurogenesis, angiogenesis, myelinogenesis, axonal growth, and apoptosis inhibition, whereas examples of detrimental effects are apoptosis induction, blood-brain barrier disruption, and demyelination. Many researches have been published on MMPs in AD. However, a comprehensive discussion of the emerging roles of MMPs and their interactions is not available. Therefore, their multiple roles in AD are discussed in this review, especially MMP-2, MMP-3, and MMP-9.

## 2. Effects of Gelatinases in AD: MMP-2 and MMP-9

Previous study has revealed that both MMP-2 and MMP-9 are induced by the presence of A*β* and highly expressed and secreted by astrocytes [3]. It has been demonstrated that MMP inhibitor II, which is reportedly highly selective for MMP-2, 9, blocks A*β*-induced release of lactate dehydrogenase in primary cultured neurons, indicating that MMP-2, 9 contribute to A*β*-induced neuronal cell death [4]. Estrogen treatment markedly increases the appearance of the active forms of both MMP-2, 9 in the culture medium, modifying MMP-9, but not MMP-2 proactive form. This is not surprising as MMP-2 is constitutively expressed at high levels in neuronal cells whereas MMP-9 is barely expressed in basal conditions but is highly inducible [5]. In the study of Merlo S, estrogens cause induction and activation of neuronal MMP-2/9, responsible in turn for enhanced A*β* degradation, highlighting an additional mechanism through which they can contribute to preserve neurons from A*β* toxicity [6]. MMP-2 is the major MMP which is directly linked to A*β* in the brain and dysfunction in this enzyme may influence the processing of A*β*_1-40/42_ in vitro and in vivo, because its deletion resulted in a greater A*β* accumulation compared with MMP-9 knockout [7]. From this it follows that MMP-2 is likely to be the major kind of gelatinase in the progression of AD.

### 2.1. MMP-2 in A*β* Pathology

MMP-2 might be assumed to have a protective role in AD. There was an increase of MMP-2 expression in astrocytes surrounding senile plaques in transgenic mice brain. MMP-2 is released in a latent form (pro-MMP-2) that requires activation by MT1-MMP. MT1-MMP was the first MMP to be identified as an integral membrane protein with a single transmembrane domain and a short cytoplasmic C-terminal tail. MT1-MMP is inhibited by the endogenous TIMP-2 and recruits pro-MMP-2 forming a ternary complex. Then, adjacent uninhibited MT1-MMP cleaves the tethered pro-MMP-2 to become MMP-2 [8].

**Figure 1:**
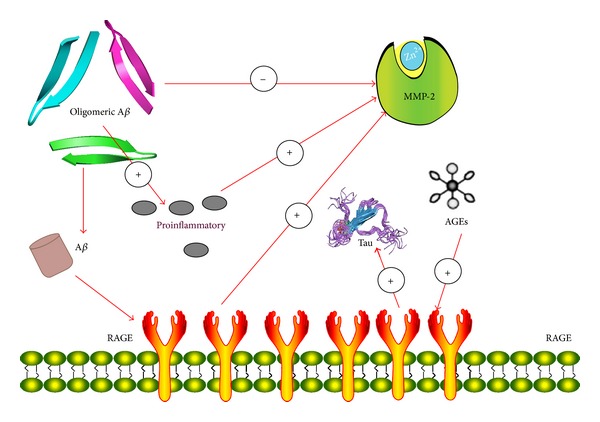
Relationship of MMP-2 and oligomeric A*β*.

Advanced glycation endproducts (AGEs), products of nonenzymatic glycation, and oxidation of proteins and lipids accumulate in diverse biological settings including inflammation, aging, and AD. AGEs play an important role in the relationship between MMP-2 and AD. Receptor for AGEs (RAGE), a multiligand receptor in the immunoglobulin superfamily, binds a broad repertoire of ligands, including AGEs, A*β*, S100/calgranulin family of proinflammatory cytokine-like mediators, and amphoterin [9]. RAGE expression in neurons and human brain microvascular endothelial cells is increased on treatment with A*β* [10]. The interaction between A*β* and RAGE triggers an intracellular signaling cascade that disrupts tight junction (TJ), leading to the breakdown of BBB integrity. By using potent inhibitors, it is confirmed that the A*β*-RAGE-CaN-MMP cascade is an important mechanism of BBB disruption and AD pathogenesis [11]. Ligand-receptor interactions of A*β* and RAGE initiate cellular signaling, leading to increased levels of MMP-2 protein, in which activation of c-Jun N-terminal kinase (JNK) MAP kinase and extracellular-signal-regulated kinases (ERK) is involved [12]. It can be proved by such two experiments as follows. Inhibitors of ERK and JNK significantly decreased A*β*-induced MMP-2 expression in brain endothelial cells (BECs). Additionally, antibody neutralization of the RAGE effectively blocked A*β*-induced activation of ERK and JNK and their contribution to elevated MMP-2 expression in BECs [12]. Furthermore, Li et al. found that AGEs could induce tau hyperphosphorylation at multiple AD-related sites and spatial memory deficits. AGEs upregulated RAGE and activated GSK-3 with inhibition of Akt, and simultaneous blockage of RAGE or inhibition of GSK-3 attenuated the AGE-induced tau hyperphosphorylation and improved the memory of the rats. AGEs inhibited LTP through RAGE/GSK-3 signaling pathway with significantly decreased levels of several postsynaptic proteins and dendritic spines [9]. It has been suggested that soluble oligomeric A*β* correlates better with dementia than insoluble fibrillar deposits, indicating that oligomeric A*β* may represent the primary toxic species in AD [13]. The soluble APP contains an inhibitor of MMP-2 and sites to interact with several components of the extracellular matrix; this secreted protein fragment is assumed to protect the extracellular matrix from the MMP-2-catalyzed degradation. The inhibitor is localized within the ISYGNDALMP sequence corresponding to residues 586–595 of APP770 and a synthetic decapeptide containing this sequence, named APP-derived inhibitory peptide (APP-IP), has MMP-2 selective inhibitory activity. This mode of interaction is probably essential for the high MMP-2 selectivity of the inhibitor because MMPs share a common architecture in the vicinity of the catalytic center, but whole structures of their substrate-binding clefts have sufficient variety for the inhibitor to distinguish MMP-2 from other MMPs [14]. The common feature of amyloid fibrils is the *β*-pleated sheet structure perpendicular to the fibril axis with a hairpin loop at the C-terminus. The conversion of soluble A*β* to fibrillar amyloid is accompanied by an increased resistance to proteolytic degradation. A*β* fibrils were observed to be more resistant to degradation by MMP-2 in AD [15]. While oligomeric A*β* directly downregulates MMP-2 expression and activation in astrocytes, it also induces production of proinflammatory cytokines, mainly in microglia surrounding amyloid plaques, including increased mRNA levels of cytokines IL1*β* and TGF-*β*, which serve as strong stimulators for MMP-2. Therefore, the ultimate outcome of the oligomeric A*β* on MMP-2 activation in astrocytes might be the combination of its direct inhibitory action on MMP-2 and the secondary action of inflammatory cytokines induced by oligomeric A*β* [8] (Figure 1).

### 2.2. Multiple Roles of MMP-9 in AD

Consideration emphasis is placed on the complicated roles of MMP-9 in AD. Expression of MMP-9 was observed in the cytoplasm of neurons, neurofibrillary tangles, senile plaques, and vascular walls of hippocampus and cerebral cortex of AD patients [16]. The proteolytic activities of MMP-2 and MMP-9 in postmortem human frontal and parietal cortical tissues obtained from subjects with a clinical diagnosis of AD, mild cognitive impairment (MCI), or no cognitive impairment were compared using gelatin zymography in the research, which demonstrated higher activity of MMP-9, but not MMP-2, in AD and MCI brains when compared to cognitively healthy brain samples. Moreover, there were inverse correlations between the Global Cognitive Score and the Mini-Mental State Examination (MMSE) score and MMP-9 activity [17]. Serum levels of MMP-9 were also found elevated in AD compared to controls and patients suffering from mild cognitive impairment [18]. Under these conditions, the increase in MMP-9 expression tends to be characteristic of AD.

**Figure 2:**
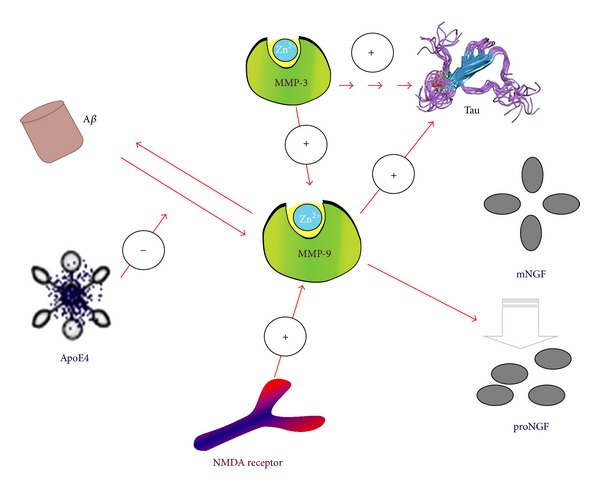
MMP-9 and MMP-3 in AD pathology.

The activation of pro-MMP-9 is controlled by a cascade of steps involving other MMPs and the plasmin system. Pro-MMP-9 can form a complex with TIMP-1, which involves interaction of the C-terminal (noninhibitory) domain of pro-MMP-9 and the C-terminal (noninhibitory) domain of TIMP-1. Moreover, low-density receptor-related protein can act as a receptor for MMP-9, which mediates internalization and degradation of the enzyme [19]. The presence of an imbalance between MMP-9 and TIMP-1 in AD patients is further supported by the association between high levels of CSF tau and high MMP-9/TIMP-1 ratios in AD group [20]. Synthesis of MMP-9 and TIMP-1 can be induced by A*β*, and the enzymes appear to play a role in senile plaque homoeostasis. N-methyl-D-aspartate (NMDA) receptors are important mediators of synaptic plasticity that are central to the neurobiological underpinnings of emotionality, learning, and memory. The cognate ligand for the NMDA receptor is glutamate, an excitatory neurotransmitter that may take part in the acceleration of AD. Activation of NMDA receptors is also involved in A*β*-induced expression of MMP-9 in vivo. A recent study identified MMP-9 as a physiologic regulator of NMDA receptor-dependent synaptic plasticity and memory. These effects of MMP-9 on NMDA receptors are mediated by an integrin *β*1-dependent pathway [21]. ApoE4 is a major genetic risk factor for AD and is associated with Down's syndrome dementia. Some other studies show that apoE4 significantly dampens A*β*-induced MMP-9 levels, possibly by downregulating the Rho-Rho kinase (ROCK) pathway, and reduction of astrocytic MMP-9 by apoE4 may affect A*β* clearance and promote A*β* deposition in AD [22, 23]. These data indicate that A*β* and NMDA receptors exert their possibly positive effects in variation of MMP-9, whereas apoE4 is reverse. The explicit mechanisms remain to be worked out (Figure 2).

MMP-9 induces A*β*, degrades A*β* and compact plaques, and is released, along with proNGF, in an activity dependent manner [24]. A*β*-induced MMP-9 involvement in BBB disruption has also been demonstrated and so does the involvement of MMP-9 in the degradation of TJ proteins, such as ZO-1 [25]. Koronyo-Hamaoui et al. found that immunization of APP/presenilin 1 double-transgenic mice with an altered myelin-derived peptide (MOG45D), loaded on dendritic cells, led to a substantial reduction of parenchymal and perivascular A*β*-plaque burden and soluble A*β*_1-42_ peptide levels as well as reduced astrogliosis and levels of a key glial scar protein (chondroitin sulphate proteoglycan). Furthermore, the levels of MMP-9, an enzyme shown to degrade A*β* and is associated with glial scar formation [26]. MMP-9, like MT1-MMP, cleaves between residues A30-I31. This site is exposed on the surface of A*β* fibrils allowing access for cleavage by MMP-9 and MT1-MMP [27]. But the increase in MMP-9 may alter the ratio from proNGF to mNGF by enhancing the degradation of mNGF in a context where pro-NGF conversion to mNGF is already diminished [27]. The molecular consequence of this shift in the balance from mNGF to proNGF may result in proapoptotic signaling via binding to the p75NTR receptor with proNGF, which could promote neuronal apoptosis perhaps involving a p75NTR/sortilin mediated mechanism [27]. Hence, increased MMP-9 and proNGF concomitant with reduced TrkA early in the progression of AD may result in a shift away from cell survival to proNGF apoptotic signaling. TrkA reduces and p75NTR activates *β* secretase cleavage of the APP, which requires NGF binding and activation of the second messenger ceramide [28]. Previous studies report that reduced cortical TrkA levels are positively associated with lower cognitive performance and increased cortical proNGF levels negatively correlate with cognitive impairment. Thus, the concomitant accumulation of cortical MMP-9 and proNGF, along with the reduction of TrkA, may represent a group of early pathobiological markers of the onset of AD [28] (Figure 2).

Pathological changes in the AD brain include amyloid-*β* (A*β*) plaques and neurofibrillary tangles, as well as neuronal death and synaptic loss. Recent neuropathologic study reports that elevated MMP-9 activity correlates with Braak stage but not with NIA-Reagan diagnosis. The major difference between Braak stage and the NIA-Reagan criteria is that the former only evaluates presence of tau pathology, whereas the latter evaluates presence of both tau and amyloid pathology [29]. Tau is a substrate of both MMP-3 and MMP-9. Increasing evidence demonstrates that small oligomers rather than the histopathologically observable large tau deposits like NFTs may be the most relevant toxic aggregate species for cells. Frost et al. recently demonstrated that proaggregatory, extracellular tau protein, can be incorporated by neurons and induce neurotoxic intracellular tau aggregation, which then spreads to other cells [29]. Sung et al. demonstrated that extracellular *α*-synuclein exerts increased cytotoxicity after cleavage with MMP-3. The elevated brain levels of MMP-3 may result in increased MMP-9 activity, since MMP-3 was demonstrated to cleave pro-MMP-9 to its active form. While MMP-3 and the nonspecific proteinases PK and trypsine reduce DMSO induced tau aggregation, cleavage by MMP-9 results in enhanced oligomer formation [30–32]. MMP-9 cleavage sites are mainly located in the N-terminal region and close to the C-terminus. MMP-9 may thus liberate the repeat domain of protein tau, facilitating oligomer formation. In accordance with this hypothesis, two possible fragments containing almost the whole repeat domain including the two hexapeptide motives were identified in all samples after limited cleavage with MMP-9, but not MMP-3, PK, or trypsine. The tau cleavage pattern of MMP-9 facilitates aggregation, while the other investigated enzymes reduce or abolish tau aggregation by degrading regions of the protein crucial to oligomer formation. Thus a proaggregatory influence on tau oligomer formation in strategic brain regions may be a potential neurotoxic side effect of MMP-9 [30] (Figure 2).

## 3. Stromelysin-1 in AD: MMP-3

MMP-3 levels are correlated to the duration of AD. MMP-3 is expressed in brain cells (astrocytes, microglia, and endothelial cells) and immune cells (T-cells and macrophages) and primarily around plaques in the parietal lobes in AD. The main physiological substrates of MMP-3 are fibronectin, laminin, and various collagens. Due to the fact that MMP-3 and MMP-10 are similar in structure and substrate specificity, one could speculate of a similar intracellular function of MMP-10 [33]. TIMP-1 is a direct inhibitor of MMP-3; and TIMP-1 has been shown to be elevated in CSF of AD patients, possibly as a reaction to the elevated MMP-3 levels [34].

MMP-3 in AD was significantly elevated in plasma and there was a trend towards increase in CSF [35]. Proteins including S100*β* and MMP-3 interacting with CSF A*β*_1-42_ may be related to A*β* brain pathology in brain atrophy, whereas proteins (including P-tau and apolipoprotein D) associated with atrophy even after adjusting for CSF A*β*_1-42_ may be related to A*β*-independent mechanisms [36]. On the other hand, genetic findings indicate a potential role of MMP-3 in the promotion of AD. In previous studies, the 5A allele of a functional polymorphism (5A/6A) of MMP-3 showed an increased risk of AD in association with the apolipoprotein E *ε*4 allele. It also has an impact on the risk of dementia in apolipoprotein E *ε*4 noncarriers [37]. Furthermore, MMP-3 is overexpressed in astrocytes and neurons exposed to A*β*_1-40_, activating a microglial response. Microglia can bind to accumulated A*β* via specific receptors, resulting in glial cell activation and transition into a proinflammatory state [38]. Microglia belongs to the family of tissue macrophages. Monocytes/macrophages are prominent cells at sites of chronic inflammation and have been shown to produce MMPs, when activated by agents such as LPS, Con A. The increase of A*β*_1-42_ by aging or genetic disorder (Alzheimer's susceptible person) may gradually upregulate the production of MMP-12 and MMP-13 by microglia and MMP-3 in astrocytes, neurons, and also microglia. By using chemical inhibitor, Ito et al. have herein shown the A*β*-induced activation of MMP-3, MMP-12, and MMP-13 to be correlated with the activation of the PI3 K/Akt signaling cascades in the microglia [39]. Accompanied with these cascades, MMP-12 has been shown to activate other MMPs such as MMP-2 and MMP-3, by which MMP12 exacerbates the cascade of proteolytic processes [39]. These findings suggest that MMP-3 takes its complicated roles in MMPs family; in other words, its effects in AD may also be double-edged.

In addition, the CSF MMP-3 correlates with the CSF T-tau and P-tau levels in the elderly controls [40]. MMP-3, PK, and trypsine may yield multiple cleavage sites in the repeat domain of tau protein, a region known to constitute the core of tau fibrils. Cleavage sites of PK and trypsine appear to be evenly distributed over the whole protein sequence, which is expected given the broad number of potential cleavage sites of these proteinases. Notably, PK and trypsine yield cleavage sites in at least one of the two hexapeptide motives known to be crucial for tau fibril formation. It can thus be speculated that cleavage by these proteinases may inhibit tau aggregation by fragmenting the protein's assembly domain [32]. Incubation of purified tau protein with active MMP-9 and MMP-3 resulted in limited proteolysis by both metalloproteinases [32]. In addition, tau fragments obtained from limited cleavage demonstrated a distinct cleavage pattern for both MMP-3 and MMP-9. MMP-3 produced multiple cleavage sites in the repeat domain of the tau protein, which is a core region of tau fibrils [32]. In contrast, MMP-9 cleavage sites were mainly located in the N-terminal region and close to the C-terminus, liberating this repeat domain of tau protein [32]. This finding suggests that the tau cleavage pattern of MMP-9 facilitates its aggregation, while MMP-3 decreases oligomer aggregation by degrading tau in regions that are crucial to the formation of fibrils. Additionally, because MMP-3 was demonstrated to be an activator of pro-MMP-9, it was suggested that the elevated brain levels of MMP-3 might result in increased MMP-9 activity and indirectly facilitate tau aggregation [32] (Figure 2).

## 4. Roles of Other MMPs and TIMPs in AD: MMP-1, MT1-MMP, and TIMP-1

Evidence has been found that enhanced MMP-1 activity in AD may contribute to the blood-brain barrier dysfunction seen in AD, but it is suggested by Lorenzl et al. that MMP-1 does not correlate with AD diagnosis or risk factors for future development of AD [18]. The MT1-MMP, which is a physiological activator of the zymogen form of MMP-2, was also expressed in reactive astrocytes in a mouse model of AD, specifically in regions with amyloid deposits [41]. More specifically, recombinant MT3-MMP showed multiple cleavage sites on A*β*PP within the A*β* domain. Mass spectrometry data showed an MT1-MMP cleavage site at the H14-Q15, which is the same as an MT3-MMP shedding site on A*β*PP. Although the shedding pattern for MT1-MMP and MT3-MMP is very similar, A*β* peptide was not degraded by recombinant MT3-MMP or by cells expressing MT3-MMP. Therefore, regarding MT-MMPs the A*β* degradation activity appears specific to MT1-MMP. MT5-MMP was also reported to have *α* secretase-like shedding activity on A*β*PP which would preclude A*β* formation.

The activities of almost all MMPs are susceptible to TIMPs inhibition, and some members of a disintegrin and a metalloproteinase family are also inhibited by these inhibitors [42]. Metalloproteinases and TIMPs form tight complexes at a 1 : 1 molar stoichiometry. There are four known mammalian TIMPs: TIMP-1, TIMP-2, TIMP-3, and TIMP-4. TIMP molecules are approximately 40% identical [43]. TIMP-1 promoted cell proliferation, and this effect was confirmed in primary cultured astrocytes induced by rTIMP-1 and A*β*_25-35_. Furthermore, the proliferative effect of A*β*_25-35_ was enhanced by the presence of TIMP-1, which suggested that TIMP-1 is mainly secreted by injured neurons and plays a role in astroglial reactivity [30]. Plasma levels of TIMP-1 and C-reactive protein in patients with AD treated with acetylcholinesterase inhibitors (AChEIs) were significantly lower than those in nontreated patients with AChEIs [44]. The potential reduction in the TIMP-1 level by AChEIs may be beneficial in AD, as elevated CSF levels of TIMP-1 have been previously detected in patients with AD [44]. Moreover, TIMPs can activate pro-MMPs. The interactions of proenzymes with TIMPs are specific: pro-MMP-2 interacts with TIMP-2, TIMP-3, or TIMP-4, whereas MMP-9 interacts with TIMP-1 or TIMP-3 [43]. However, their functional significance is still unclear, with the exception of the pro-MMP-2-TIMP-2 complex. The pro-MMP-2/TIMP-2 complexes do not involve the N-terminal domain of the inhibitory molecule; thus, the complexes have the ability to interact with a second MMP molecule. This activation is completed by an autolytic cleavage by MMP-2 [43].

## 5. Potential Values of MMPs as Biomarkers in AD Diagnosis

AD is often clinically diagnosed far after significant synaptic and neuronal loss has already occurred. The examination of postmortem brain tissues from patients with AD and pathological findings such as neurofibrillary tangles and senile plaques (as well as the expression of MMPs) only provides a postmortem confirmation of the diagnosis of AD. The identification of protein markers in biological fluids, such as plasma or CSF, could help diagnose AD at an early stage to allow for therapeutic intervention. In particular, the identification of blood biomarkers of AD is ideal, because this type of sample is easy to access and less invasive to obtain in patients.

In one study, assessment of the concentrations of various metalloproteinases and AD biomarkers of A*β*_1-42_, T-tau, and P-tau181, which were assayed simultaneously using luminex ELISA technology in the CSF, revealed significant decreases in MMP-2 and MMP-3 levels in the CSF in the samples with significantly reduced A*β* levels [45]. In the group of healthy elderly individuals, Stomrud et al. observed that the individuals with risk markers for possible future AD, that is, AD-supportive CSF biomarkers (tau and A*β*_1-42_) or presence of the APOE *ε*4 allele which is one gene shown to increase the risk of developing AD, have higher CSF MMP-3 and MMP-9 levels and a higher CSF MMP-3/TIMP-1 ratio compared with the individuals without risk markers [40]. It has been proved in CSF by multiplexing techniques that concentrations of MMP-9 and TIMP-1 in AD patients were significantly lower than those in the subcortical vascular disease group, whereas MMP-10 levels were significantly higher in AD patients compared to healthy controls. After determining protein levels in the CSF, data analysis was performed using a multivariate discriminant algorithm, which identified specific CSF concentrations of MMP-2, 9 and TIMP-1 as the proteins that contributed the most to the separation between SVD and AD, with high sensitivity (89%), specificity (90%), and area under the ROC curve (0.92) [19]. A comparison of VaD with AD patients revealed significantly higher CSF levels of MMP-9 activity in VaD patients compared to those with AD or control subjects. Activities of MMP-2 and the concentrations of TIMP-1 and TIMP-2 were similar in both patient groups and controls [46]. Based on our expertise in this field and literature, MMPs cannot be applied for the routine diagnostics of AD yet. Implementation of the determination of these proteins requires development of immunoenzymatic tests that would be applicable on commonly used laboratory platforms for in vitro diagnostics, not only for scientific research.

## 6. Conclusions

Taken together, MMPs exert multiple effects in AD. In addition to the function of MMPs in AD states, these enzymes have many roles in physiological processes, such as angiogenesis and neurogenesis. In the majority situation, their confused traits are unpredictable. Therefore, the clinical utility of these proteins, particularly in plasma or serum, as potential early diagnostic biomarkers for AD, remains to be established. To date, a large number of MMP inhibitors based on hydroxamic acid derivatives or other synthetic inhibitors have been designed. However, none of them has been developed successfully as anti-AD drugs mainly because of deleterious side effects; the broad specificity of the MMP inhibitors must be a stiff obstacle for developing safe and effective drugs. The dual action of MMPs complicates efforts at treatment with broad-spectrum MMP inhibitors. More research is needed to understand the diverse roles of these proteases to design specific drugs and devise therapeutic strategies for AD.

## References

[1] Ethell IM, Ethell DW (2007). Matrix metalloproteinases in brain development and remodeling: Synaptic functions and targets. *Journal of Neuroscience Research*.

[2] Mroczko B, Groblewska M, Barcikowska M (2013). The role of matrix metalloproteinases and tissue inhibitors of metalloproteinases in the pathophysiology of neurodegeneration: a literature study. *Journal of Alzheimer's Disease*.

[3] Fujimoto M, Takagi Y, Aoki T (2008). Tissue inhibitor of metalloproteinases protect blood-brain barrier disruption in focal cerebral ischemia. *Journal of Cerebral Blood Flow and Metabolism*.

[4] Haorah J, Ramirez SH, Schall K, Smith D, Pandya R, Persidsky Y (2007). Oxidative stress activates protein tyrosine kinase and matrix metalloproteinases leading to blood-brain barrier dysfunction. *Journal of Neurochemistry*.

[5] Sbai O, Ferhat L, Bernard A (2008). Vesicular trafficking and secretion of matrix metalloproteinases-2, -9 and tissue inhibitor of metalloproteinases-1 in neuronal cells. *Molecular and Cellular Neuroscience*.

[6] Merlo S, Sortino MA (2012). Estrogen activates matrix metalloproteinases-2 and -9 to increase beta amyloid degradation. *Molecular and Cellular Neuroscience*.

[7] Miners JS, Baig S, Palmer J, Palmer LE, Kehoe PG, Love S (2008). A*β*-degrading enzymes in Alzheimer's disease. *Brain Pathology*.

[8] Li W, Poteet E, Xie L, Liu R, Wen Y, Yang S (2011). Regulation of matrix metalloproteinase 2 by oligomeric amyloid *β* protein. *Brain Research*.

[9] Li X-H, Lv B-L, Xie J-Z, Liu J, Zhou X-W, Wang J (2012). AGEs induce Alzheimer-like tau pathology and memory deficit via RAGE-mediated GSK-3 activation. *Neurobiology of Aging*.

[10] Cho HJ, Son SM, Jin SM (2009). RAGE regulates BACE1 and A*β* generation via NFAT1 activation in Alzheimer's disease animal model. *The FASEB Journal*.

[11] Kook S, Hong HS, Moon M, Ha CM, Chang S, Mook-Jung I (2012). A*β*
_1-42_-rage interaction disrupts tight junctions of the blood-brain barrier via Ca^2+^-calcineurin signaling. *Journal of Neuroscience*.

[12] Du H, Li P, Wang J, Qing X, Li W (2012). The interaction of amyloid b and the receptor for advanced glycation endproducts induces matrix metalloproteinase-2 expression in brain endothelial cells. *Cellular and Molecular Neurobiology*.

[13] Haass C, Selkoe DJ (2007). Soluble protein oligomers in neurodegeneration: lessons from the Alzheimer's amyloid *β*-peptide. *Nature Reviews Molecular Cell Biology*.

[14] Hashimoto H, Takeuchi T, Komatsu K, Miyazaki K, Sato M, Higashi S (2011). Structural basis for matrix metalloproteinase-2 (MMP-2)-selective inhibitory action of *β*-amyloid precursor protein-derived inhibitor. *The Journal of Biological Chemistry*.

[15] Crouch PJ, Tew DJ, Du T (2009). Restored degradation of the Alzheimer's amyloid-*β* peptide by targeting amyloid formation. *Journal of Neurochemistry*.

[16] Mizoguchi H, Takuma K, Fukuzaki E (2009). Matrix metalloprotease-9 inhibition improves amyloid *β*-mediated cognitive impairment and neurotoxicity in mice. *Journal of Pharmacology and Experimental Therapeutics*.

[17] Bruno MA, Mufson EJ, Wuu J, Cuello AC (2009). Increased matrix metalloproteinase 9 activity in mild cognitive impairment. *Journal of Neuropathology and Experimental Neurology*.

[18] Lorenzl S, Buerger K, Hampel H, Beal MF (2008). Profiles of matrix metalloproteinases and their inhibitors in plasma of patients with dementia. *International Psychogeriatrics*.

[19] Bjerke M, Zetterberg H, Edman Å, Blennow K, Wallin A, Andreasson U (2011). Cerebrospinal fluid matrix metalloproteinases and tissue inhibitor of metalloproteinases in combination with subcortical and cortical biomarkers in vascular dementia and Alzheimer's disease. *Journal of Alzheimer's Disease*.

[20] Talamagas AA, Efthimiopoulos S, Tsilibary EC, Figueiredo-Pereira ME, Tzinia AK (2007). Abeta(1–40)-induced secretion of matrix metalloproteinase-9 results in sAPP*α* release by association with cell surface APP. *Neurobiology of Disease*.

[21] Michaluk P, Mikasova L, Groc L, Frischknecht R, Choquet D, Kaczmarek L (2009). Matrix metalloproteinase-9 controls NMDA receptor surface diffusion through integrin *β*1 signaling. *Journal of Neuroscience*.

[22] Guo S, Wang S, Kim WJ (2006). Effects of apoE isoforms on beta-amyloid-induced matrix metalloproteinase-9 in rat astrocytes. *Brain Research*.

[23] Bell RD, Winkler EA, Singh I (2012). Apolipoprotein e controls cerebrovascular integrity via cyclophilin A. *Nature*.

[24] Yan P, Hu X, Song H (2006). Matrix metalloproteinase-9 degrades amyloid-*β* fibrils in vitro and compact plaques in situ. *The Journal of Biological Chemistry*.

[25] Barkus C, McHugh SB, Sprengel R, Seeburg PH, Rawlins JNP, Bannerman DM (2010). Hippocampal NMDA receptors and anxiety: at the interface between cognition and emotion. *European Journal of Pharmacology*.

[26] Koronyo-Hamaoui M, Ko MK, Koronyo Y (2009). Attenuation of AD-like neuropathology by harnessing peripheral immune cells: local elevation of IL-10 and MMP-9. *Journal of Neurochemistry*.

[27] Clewes O, Fahey MS, Tyler SJ (2008). Human ProNGF: biological effects and binding profiles at TrkA, P75^NTR^ and sortilin. *Journal of Neurochemistry*.

[28] Costantini C, Weindruch R, Della Valle G, Puglielli L (2005). A TrkA-to-p75^NTR^ molecular switch activates amyloid *β*-peptide generation during aging. *Biochemical Journal*.

[29] Frost B, Jacks RL, Diamond MI (2009). Propagation of tau misfolding from the outside to the inside of a cell. *The Journal of Biological Chemistry*.

[30] Hernández-Guillamon M, Delgado P, Ortega L (2009). Neuronal TIMP-1 release accompanies astrocytic MMP-9 secretion and enhances astrocyte proliferation induced by *β*-amyloid 25-35 fragment. *Journal of Neuroscience Research*.

[31] Mannello F, Medda V (2012). Nuclear localization of Matrix metalloproteinases. *Progress in Histochemistry and Cytochemistry*.

[32] Nübling G, Levin J, Bader B (2012). Limited cleavage of tau with matrix-metalloproteinase MMP-9, but not MMP-3, enhances tau oligomer formation. *Experimental Neurology*.

[33] Choi DH, Kim E, Son HJ (2008). A novel intracellular role of matrix metalloproteinase-3 during apoptosis of dopaminergic cells. *Journal of Neurochemistry*.

[34] Reitz C, van Rooij FJA, Soares HD (2010). Matrix metalloproteinase 3 haplotypes and plasma amyloid beta levels: the Rotterdam study. *Neurobiology of Aging*.

[35] Horstmann S, Budig L, Gardner H (2010). Matrix metalloproteinases in peripheral blood and cerebrospinal fluid in patients with Alzheimer's disease. *International Psychogeriatrics*.

[36] Mattsson N, Insel P, Nosheny R (2014). Effects of cerebrospinal fluid proteins on brain atrophy rates in cognitively healthy older adults. *Neurobiology of Aging*.

[37] Helbecque N, Cottel D, Hermant X, Amouyel P (2007). Impact of the matrix metalloproteinase MMP-3 on dementia. *Neurobiology of Aging*.

[38] Vasto S, Candore G, Duro G, Lio D, Grimaldi MP, Caruso C (2007). Alzheimer's disease and genetics of inflammation: a pharmacogenomic vision. *Pharmacogenomics*.

[39] Ito S, Kimura K, Haneda M, Ishida Y, Sawada M, Isobe K (2007). Induction of matrix metalloproteinases (MMP3, MMP12 and MMP13) expression in the microglia by amyloid-*β* stimulation via the PI3K/Akt pathway. *Experimental Gerontology*.

[40] Stomrud E, Björkqvist M, Janciauskiene S, Minthon L, Hansson O (2010). Alterations of matrix metalloproteinases in the healthy elderly with increased risk of prodromal Alzheimer's disease. *Alzheimer's Research and Therapy*.

[41] Liao M, van Nostrand WE (2010). Degradation of soluble and fibrillar amyloid *β*-protein by matrix metalloproteinase (MT1-MMP) in vitro. *Biochemistry*.

[42] Higashi S, Miyazaki K (2008). Identification of amino acid residues of the matrix metalloproteinase-2 essential for its selective inhibition by *β*-amyloid precursor protein-derived inhibitor. *The Journal of Biological Chemistry*.

[43] Brew K, Nagase H (2010). The tissue inhibitors of metalloproteinases (TIMPs): an ancient family with structural and functional diversity. *Biochimica et Biophysica Acta—Molecular Cell Research*.

[44] Inspector M, Aharon-Perez J, Glass-Marmor L, Miller A (2005). Matrix metalloproteinase-9, its tissue inhibitor (TIMP)-1 and CRP in Alzheimer's disease. *European Neurology*.

[45] Lewczuk P, Popp J, Lelental N (2012). Cerebrospinal fluid soluble amyloid-*β* protein precursor as a potential novel biomarkers of Alzheimer's disease. *Journal of Alzheimer's Disease*.

[46] Adair JC, Charlie J, Dencoff JE (2004). Measurement of gelatinase B (MMP-9) in the cerebrospinal fluid of patients with vascular dementia and Alzheimer disease. *Stroke*.

